# A Critical Role for ZDHHC2 in Metastasis and Recurrence in Human Hepatocellular Carcinoma

**DOI:** 10.1155/2014/832712

**Published:** 2014-06-09

**Authors:** Chuanhui Peng, Zhijun Zhang, Jian Wu, Zhen Lv, Jie Tang, Haiyang Xie, Lin Zhou, Shusen Zheng

**Affiliations:** ^1^Key Laboratory of Combined Multi-Organ Transplantation, Ministry of Public Health, Hangzhou, Zhejiang 310003, China; ^2^Division of Hepatobiliary and Pancreatic Surgery, Department of Surgery, First Affiliated Hospital, School of Medicine, Zhejiang University, Hangzhou, Zhejiang 310003, China

## Abstract

It has been demonstrated that loss of heterozygosity (LOH) was frequently observed on chromosomes 8p22-p23 in hepatocellular carcinoma (HCC) and was associated with metastasis and prognosis of HCC. However, putative genes functioning on this chromosomal region remain unknown. In this study, we evaluated LOH status of four genes on 8p22-p23 (MCPH1, TUSC3, KIAA1456, and ZDHHC2). LOH on ZDHHC2 was associated with early metastatic recurrence of HCC following liver transplantation and was correlated with tumor size and portal vein tumor thrombi. Furthermore, our results indicate that ZDHHC2 expression was frequently decreased in HCC. Overexpression of ZDHHC2 could inhibit proliferation, migration, and invasion of HCC cell line Bel-7402 *in vitro*. These results suggest an important role for ZDHHC2 as a tumor suppressor in metastasis and recurrence of HCC.

## 1. Introduction

Hepatocellular carcinoma (HCC) is one of the most common human malignancies worldwide, with a high rate of incidence and mortality [1, 2]. Over the past decades, many efforts have been made to improve the overall survival rate of HCC, of which both liver transplantation (LT) and surgical resection are considered potentially curative treatments for early-stage HCC. However, the long-term survival of HCC patients after surgery still remains poor due to the frequent metastasis and recurrence. Therefore, it is essential to explore the molecular pathogenesis of HCC metastasis and recurrence and detect novel molecular targets in HCC.

Loss of heterozygosity (LOH) refers to the loss of one of the two alleles at one or more loci in a heterozygote [3]. LOH in a chromosomal fragment implies the presence of putative tumor suppressor genes (TSGs) and can be used as an indirect way to identify TSGs [4]. Inactivation of TSGs was thought to be correlated with the metastasis and recurrence of primary cancer [5, 6]. Previous reports have revealed that frequent allelic losses on chromosomes 1p, 4q, 6q, 8p, 9p, 10q, 11p, 13q, 14q, 16q, and 17p are commonly observed in HCC patients [3, 4, 7–10]. Notably, loss of 8p is one of the most frequent chromosomal alterations in a variety of cancers including HCC [11–16]. Several studies have demonstrated that deletions of allele on 8p22-p23 are associated with metastasis and prognosis of HCC [7, 11, 17]. This suggests that 8p22-p23 might harbor one or more TSGs that are important in the progression, especially in the metastasis and recurrence of HCC. Until now, only few critical TSGs have been found on 8p22-p23, and it is necessary to investigate the LOH status of candidate genes in order to identify TSGs within this region. Additionally, studies about the roles of LOH in progression and prognosis of HCC in patients with LT are limited in number.

In this study, we selected the four most noteworthy genes on 8p22-p23 (MCPH1, TUSC3, KIAA1456, and ZDHHC2) to reveal the potential relationships between LOH of these genes and the clinical characteristics in HCC after LT. We found that the LOH on ZDHHC2 was associated with early metastatic recurrence of HCC following LT and was correlated with tumor size and portal vein tumor thrombi (PVTT). Further study provides the evidence that ZDHHC2 has important role as a tumor suppressor in metastasis and recurrence of HCC.

## 2. Materials and Methods

### 2.1. Clinical Specimens

This study involved three independent cohorts of HCC patients. All 3 cohorts were from the First Affiliated Hospital, Zhejiang University School of Medicine, Zhejiang, China. Cohort 1 was a total of 40 randomly selected HCC patients with LT performed during 2006 and 2009; cohort 2 and cohort 3 were, respectively, a total of 55 and 23 HCC patients following partial hepatectomy performed during 2010 and 2011. This study was approved by the Ethical Review Committee of the First Affiliated Hospital, Zhejiang University School of Medicine, and informed consent was obtained according to the Declaration of Helsinki.

### 2.2. Sample Collection

Primary HCC and adjacent normal liver tissues were obtained during the operation. All tissue samples were immediately cut into small pieces, snap-frozen in liquid nitrogen, and stored at −80°C. Diagnosis for all samples was confirmed by histopathological examination. Genomic DNA was extracted using the QIAamp DNA Mini Kit (Qiagen, Valencia, CA) in accordance with the manufacturer's instructions.

### 2.3. SNP-Based LOH Analysis

**Figure 1:**
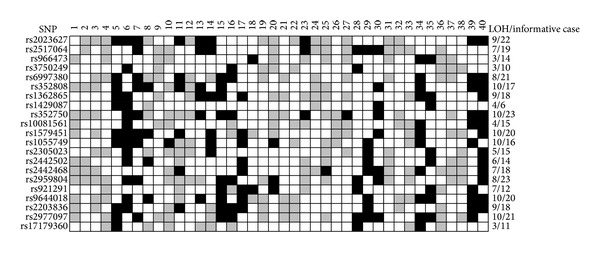
Status and frequency of LOH on each SNP in 40 HCC cases. Black box: LOH; gray box: retention; white box: noninformative case.

Five SNPs (rs2023627, rs2517064, rs966473, rs3750249, and rs6997380) that resided in ZDHHC2, six SNPs (rs352808, rs1362865, rs1429087, rs352750, rs10081561, and rs1579451) in TUSC3, six SNPs (rs1055749, rs2305023, rs2442502, rs2442468, rs2959804, and rs921291) in MCPH1, and four SNPs (rs9644018, rs2203836, rs2977097, and rs17179360) in KIAA1456 were selected to determine LOH. Sequences of these SNPs were obtained from the Human SNP Database. The methods for multiplex polymerase chain reaction (PCR) and LOH analysis were described previously [3], and SNPs were amplified under the same PCR conditions as microsatellite markers. Information for each SNP on these candidates is shown in Figure 1. LOH was defined as the value of LOH index within a range between 0.7 and 1.5 [18]. LOH on a single gene was determined to be positive when one or more SNPs on each gene detected LOH.

### 2.4. qRT-PCR

Total RNA from HCC samples was isolated using Trizol reagent (Invitrogen, Carlsbad, CA, USA) following the manufacturer's instructions; then cDNA was synthesized. qPCR reactions were performed by the ABI7500 system (Applied Biosystems, CA) and SYBR green dye (TaKaRa Biotechnology, Dalian, China). GAPDH was used as an internal control. The primers' sequences used in this study were listed in Supplementary Table  1 available online at http://dx.doi.org/10.1155/2014/832712. Every sample was tested in duplicate.

### 2.5. Immunohistochemistry

Immunohistochemistry (IHC) of paraffin-embedded tissue sections was performed using the antibody ZDHHC2 (AP5592a, Abgent, CA, USA) as we have previously described [19]. Evaluation and semiquantitative estimation of IHC results were performed independently by two pathologists without knowledge of the clinicopathological outcomes. Intensity was graded as 0 (no staining), 1 (weak staining), 2 (moderate staining), or 3 (strong staining). The abundance of positive cells was graded from 0 to 4 (0, <5% positive cells; 1, 5–25%; 2, 26–50%; 3, 51–75%; 4, >75%). The composite score obtained by multiplying the two values was analyzed.

### 2.6. Cell Culture

Normal liver cell line L-02; liver cancer cell lines Hep3B, HuH-7, and Bel-7402; and the metastasis-capable human HCC cell lines MHCC97L and HCCLM3 were purchased from American Type Culture Collection (Manassas, VA), Shanghai Institute of Cell Biology (Shanghai, China), and Liver Cancer Institute of Fudan University (Shanghai, China). All of the cell lines were maintained in the recommended culture condition and incubated at 37°C humidified environment containing 5% CO_2_.

### 2.7. Overexpression of ZDHHC2

The plasmid of the coding sequence (CDS) length ZDHHC2 with EGFP-tag and the negative control plasmid only containing EGFP-tag were completed by the Genechem Company (Shanghai, China). A PCR-amplified CDS-length ZDHHC2 fragment was inserted into the XhoI/KpnI sites of GV230 vector (Genechem, Shanghai, China), using the primers forward, 5′-TCCGCTCGAGATGGCGCCCTCGGGCC-3′ and reverse, 5′-ATGGGGTACCGTAGTCTCATTTTCCATGGTTAATG-3′. Transfection of the vectors was performed with Lipofectamine 2000 (Invitrogen, Carlsbad, CA, USA) following the manufacturer's instructions.

### 2.8. Western Blotting Analysis

Protein extraction and western blotting analysis were performed as previously described [20]. The following antibodies were used: anti-ZDHHC2, SantaCruz (sc-292338); anti-b-actin, Sigma-Aldrich (A1978); anti-GFP-Tag, Abmart (M20004).

### 2.9. Cell Proliferation Assay

Cell growth was determined by Cell Counting Kit-8 (CCK-8) cell proliferation assay (Dojindo Laboratories, Kumamoto, Japan) according to the manufacturer's instructions as previously described [21].

### 2.10. Transwell Migration and Invasion Assay

For invasion assay, 48 hours after transfection, 5 × 10*E*4 Bel-7402 cells in serum-free RPMI1640 were seeded into the upper chambers of each well (24-well insert, 8-mm pore size, Millipore, Billerica, MA, USA) coated with Matrigel (BD Bioscience, San Jose, CA, USA). For migration assay, the upper chambers were not coated with Matrigel, and cells were seeded after 24-hour transfection. RPMI1640 containing 10% FBS was placed in the lower chambers as a chemoattractant. After 24 hours of incubation, cells on the upper membrane surface were wiped off, and the cells that invaded across the Matrigel membrane were fixed with 100% methanol and stained with 0.2% crystal violet. The number of invasive cells was then counted (five randomly chosen high-power fields for each membrane) under a microscope.

### 2.11. Statistical Analysis

Comparisons between LOH and clinicopathological data were analyzed using chi-square test or Fisher exact test. Cumulative recurrence-free survival was assessed by Kaplan-Meier method and univariate analysis by log-rank test. Variables were brought into multivariate analysis when there was statistical significance in univariate analysis. Multivariate analysis was assessed using Cox proportional hazard model to identify variables that were independent predictors of clinical outcome. Independent Student's *t*-test was used to analyze the differences between two groups. Data are presented as the mean±SD, unless otherwise indicated. *P* < 0.05 was considered statistically significant. All statistical analyses were performed with the use of SPSS version 16.0 software (SPSS, Chicago, IL).

## 3. Results

### 3.1. LOH of ZDHHC2 Associates with Recurrence of Hepatocellular Carcinoma following Liver Transplantation

DNA from forty individuals with HCC who had received a liver transplants was analyzed for twenty-one SNPs of these four specific genes. Their mean heterozygosity was 87.5%. The heterozygosity on ZDHHC2, MCPH1, TUSC3, and KIAA1456 was correspondingly 95% (38 of 40), 92.5% (37 of 40), 85% (34 of 40), and 77.5% (31 of 40). Distribution of LOH on each SNP is shown in Figure 1. The LOH frequencies at all selected genes were so high, with a mean value of 50.3%. The frequencies of LOH on ZDHHC2, MCPH1, TUSC3, and KIAA1456 were 45% (17 of 38), 54% (20 of 37), 50% (17 of 34), and 52% (16 of 31), respectively.

To study the association of LOH on each gene with clinicopathological characteristics and to explore its potential biological role in HCC initiation, development, and progression, we compared frequencies of LOH based on clinicopathological findings including age, preoperative serum AFP level, tumor number, tumor size, PVTT and histopathologic grading. All of the results between clinicopathological features and LOH in HCC were shown in Table 1.

**Figure 2:**
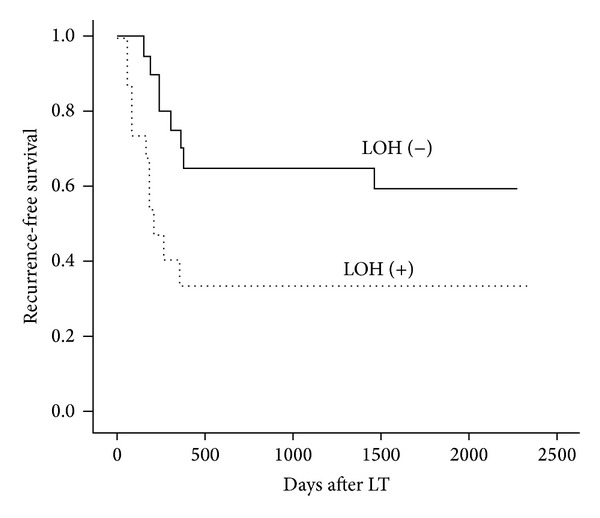
Kaplan-Meier analysis and log-rank test of the recurrence-free survival according to the LOH status of ZDHHC2 in 38 informative cases.

To determine the association between gene LOH status and prognostic data, we performed univariate and multivariate survival analysis. As a result, we found that the 1-year cumulative recurrence-free survival in HCC patients with LOH on ZDHHC2 was significantly lower than that with heterozygosity retention (*P* = 0.022, Figure 2). However, there were no significant differences between recurrence-free survival and LOHon MCPH1 (*P* = 0.320), TUSC3 (*P* = 0.546), and KIAA1456 (*P* = 0.564). Furthermore, based on the univariate analysis, we investigate the relationship between 1-year cumulative recurrence-free survival and clinicopathological factors. Taken together, significant associations were found between HCC recurrence and the following variables: LOH on ZDHHC2, preoperative AFP level >400 ng/mL, tumor size >5 cm, and presence of PVTT (Table 2). Factors which were found to have prognostic value including the clinicopathologic characteristics and gene LOH status in the univariate analysis were added into the corresponding multivariate Cox model of survival, and only PVTT was identified as independent prognostic factor for recurrence-free survival in HCC patients after LT (Table 3).

### 3.2. Decreased Expression of ZDHHC2 Is Observed in HCC Tissue Samples and HCC Cell Lines

**Figure 3:**
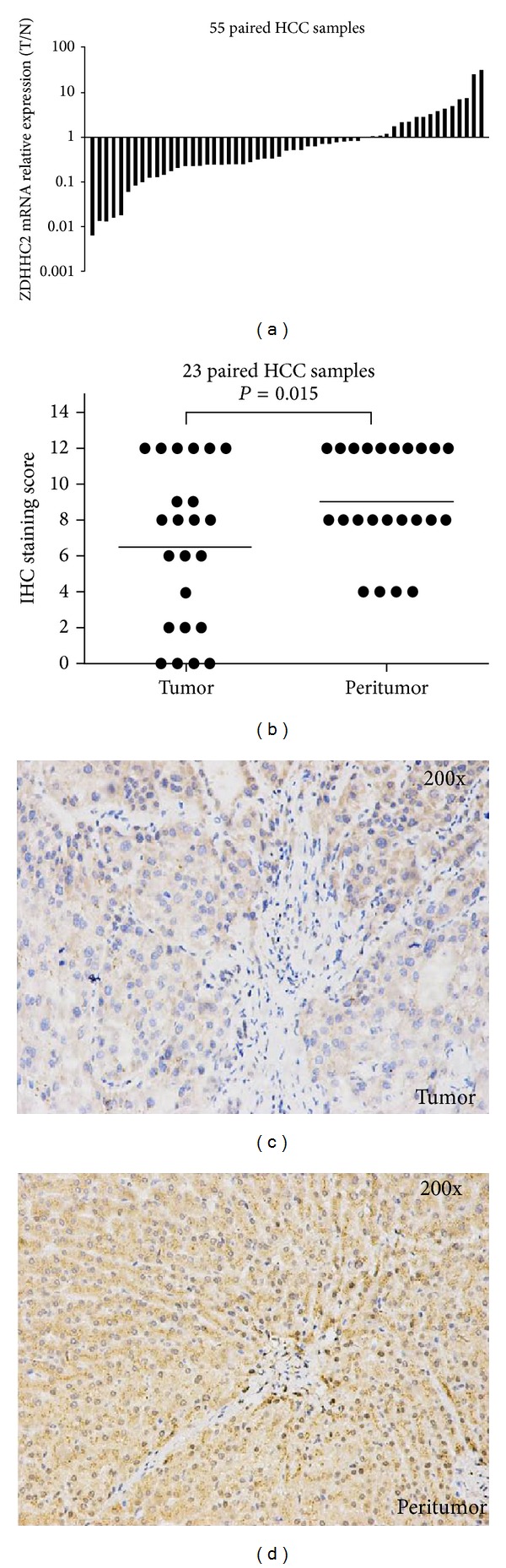
Frequently decreased expression of ZDHHC2 in human HCC tissues. (a) In cohort 2 (55 paired HCC tissue samples), the expression levels of ZDHHC2 mRNA are significantly lower in tumor tissues than peritumor tissues from the same patients (*P* = 0.003). (b) Relative IHC staining of ZDHHC2 expression in paired HCC tissue samples (cohort 3, *n* = 23). The ZDHHC2 expression level was significantly downregulated in tumors compared with the corresponding adjacent nontumor liver tissues (*P* = 0.015). (c) and (d) are preventative pictures, respectively (200x).

To investigate the expression of ZDHHC2 in tumor tissues and peritumor tissues from HCC samples, we conducted qRT-PCR in cohort 2 of 55 HCC samples and IHC in cohort 3 of 23 HCC samples. We found that mRNA level of ZDHHC2 expression is significantly lower in tumor tissues than peritumor tissues from the same patients (*P* = 0.003) (Figure 3(a)). We analyzed the correlation between ZDHHC2 mRNA expression and clinical parameters including age, preoperative serum AFP level, tumor number, tumor size, PVTT, and histopathologic grading. In accord with the correlation of LOH of ZDHHC2 and clinical parameters, lower expression of ZDHHC2 was associated with the tumor size and the presence of PVTT (Supplementary Table  2).

Furthermore, by multiplying the values of staining intensity and relative abundance of positive cells, we found that the mean staining score of ZDHHC2 in tumor tissues is significantly lower than those in peritumor tissues (*P* = 0.015) (Figure 3(b)). The representative pictures of tumors and peritumors are shown in Figures 3(c) and 3(d).

**Figure 4:**
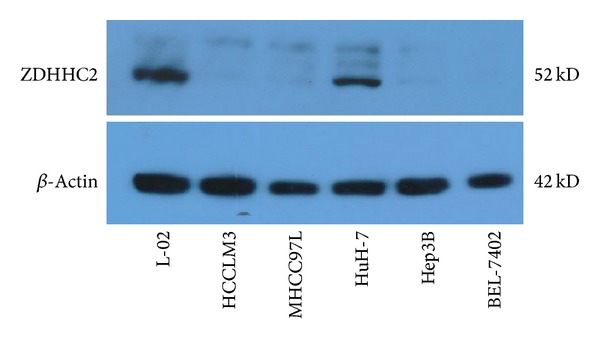
ZDHHC2 expression levels in HCC cell lines were detected by western blot. Compared to normal liver cell line L-02, ZDHHC2 expression level in HCC cell lines was significantly lower.

Moreover, ZDHHC2 expression level in HCC cell lines was detected by western blotting. Compared to normal liver cell line L-02, ZDHHC2 expression levels in HCC cell lines (Hep3B, HuH-7, Bel-7402, MHCC97L, and HCCLM3) were significantly lower (Figure 4).

### 3.3. Overexpression of ZDHHC2 Inhibits Cell Proliferation and Invasion in BEL-7402* In Vitro*

**Figure 5:**
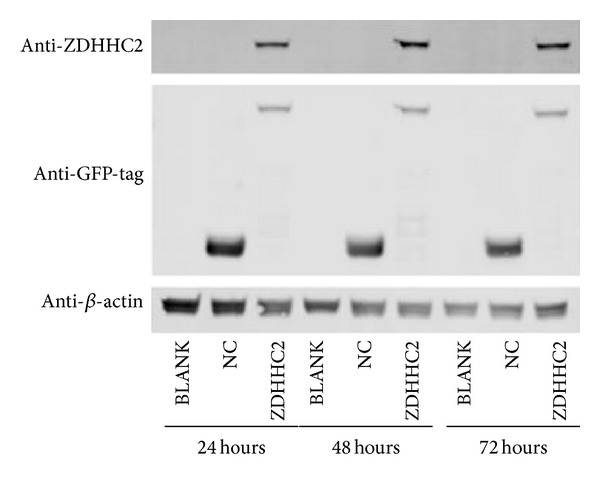
Plasmids containing ZDHHC2-EGFP or EGFP were transfected into Bel-7402. The transfection was monitored by western blotting analysis at the 24-, 48-, and 72-hour time points. Bel-7402 without any treatment (BLANK), transiently transfected with negative control vector (NC), and transiently transfected with overexpression vector (ZDHHC2).

To determine whether reduction of ZDHHC2 affects biological function in HCC and overexpression of ZDHHC2 could be used as potential consideration to treat HCC, further experimental studies were then performed. Overexpression vector and negative control vector are transiently transfected into Bel-7402 HCC cell line, then downstream analysis was performed. Transient transfection was successfully performed as shown in western blotting analysis (Figure 5). ZDHHC2 overexpression lasts stably at 24-, 48-, and 72-hour time points.

**Figure 6:**
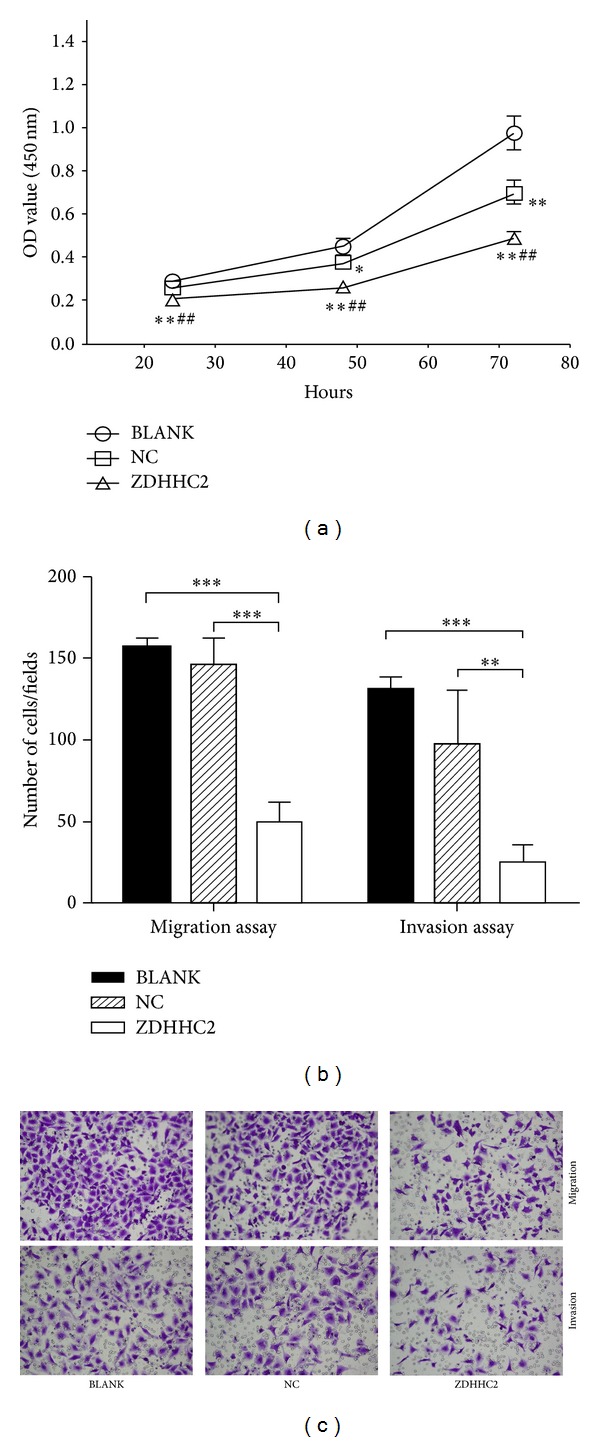
CCK-8 cell proliferation assay and transwell migration and invasion assay were conducted in Bel-7402 without any treatment (BLANK), transiently transfected with negative control vector (NC), and transiently transfected with overexpression vector (ZDHHC2). (a) Overexpression of ZDHHC2 significantly impaired Bel-7402 proliferation (*n* = 3, *P < 0.05 compared to BLANK group, **P < 0.01 compared to BLANK group, and ^##^ *P* < 0.01 compared to NC group). (b) Overexpression of ZDHHC2 inhibits the migration and invasion of BEL-7402 cell (*n* = 5, **P < 0.01, and ***P < 0.001) compared to both BLANK group and NC group. Representative pictures are shown in (c).

CCK-8 cell proliferation assay and transwell migration and invasion assay were conducted in Bel-7402 without any treatment (BLANK), transiently transfected with negative control vector (NC), and transiently transfected with overexpression vector (ZDHHC2). The results showed that overexpression of ZDHHC2 significantly impaired the proliferation (Figure 6(a)) and migratory and invasive capacity of Bel-7402 cell (Figures 6(b) and 6(c)) compared to both BLANK group and NC group.

## 4. Discussion

HCC is a frequent and highly aggressive malignancy worldwide with very poor prognosis. Recently, LT has been recognized as a potential curative treatment for early-stage HCC patients. However, the clinical outcome remains challenging because of a high incidence of tumor recurrence after LT [22]. Recurrence is principally attributable to the presence of microscopic extrahepatic metastatic foci before LT [23], which is also known as metastastic recurrence. A better understanding of the underlying molecular mechanisms governing cancer metastastic recurrence will be a great help for predicting prognosis.

Numerous studies have revealed a high-LOH frequency on 8p22-p23 in HCC [7, 9, 17, 24]. Our previous report has also supported [3]. This implies that one or several TSGs may lie within this region. Therefore, the genes on 8p22-p23 are widely investigated and several new genes have been identified as TSGs in recent years, such as CUB and Sushi multiple domains 1 (CSMD1) [9], deleted in liver cancer 1 (DLC1) [25] and mitochondrial tumour suppressor 1 (MTUS1) [26].

In the current study, we confirmed that LOH was a prevalent event on 8p22-p23 in HCC, with frequencies of LOHs on four specific genes (MCPH1, KIAA1456, TUSC3, and ZDHHC2) ranging from 45% to 54%. Interestingly, LOH on ZDHHC2 was clearly associated with early metastastic recurrence of HCC following LT, although it had the lowest LOH frequency of these four genes. In all 38 informative cases, patients with LOH had an increasing risk of early metastastic recurrence post-LT, which directly linked to patients' long-term survival. Multivariate analyses revealed that LOH on ZDHHC2 could predict the risk of HCC early recurrence together with AFP level, tumor size, and PVTT but was not an independent prognostic factor. In addition, our data showed that LOH status of ZDHHC2 was correlated with some clinicopathological parameters, including tumor size and PVTT. These data suggested that LOH on ZDHHC2 may serve as a molecular event in advanced HCC, as tumor size >5 cm and PVTT were representatives of advancement of HCC.

ZDHHC2 was originally described as REAM (reduced expression in metastasis), because its silence was associated with increased metastatic potential of cancer cells [27]. It was reported that reduced ZDHHC2 expression is observed in gastric adenocarcinoma patients and associated with lymph node metastasis and independently predicts an unfavorable prognosis [28]. However, the expression level of ZDHHC2 in HCC remains to be explored. Our study provides the evidence that ZDHHC2 expression was frequently decreased in HCC. Overexpression of ZDHHC2 could inhibit proliferation, migration, and invasion of HCC cell line Bel-7402* in vitro*. These results suggest an important role for ZDHHC2 as a tumor suppressor in metastasis and recurrence of HCC.

DHHC2, encoded by ZDHHC2, is one member of a family of more than 20 palmitoyl acyltransferases (PATs) characterized by an Asp-His-His-Cys (DHHC) motif [29]. Protein palmitoylation, mediated by PATs, can affect proteins in many ways, including regulating membrane attachment, subcellular trafficking, and membrane microlocalisation [29]. Many DHHC2-substrate interactions have been identified so far, including PSD95, SNAP25, SNAP23, CKAP4, CD9, and CD151 [29]. The decrease or absence of DHHC2 expression could influence the palmitoylation of these substrates, and whatever role palmitoylation had in signaling downstream from that event would be disrupted. Of these substrates, CKAP4, CD9, and CD151 have been associated with initiation and progression of cancer. Decreased expression of DHHC2 leads to reduced palmitoylation of CKAP4, making it no longer traffick efficiently to the cell surface where it act as a receptor for antiproliferative factor (APF) [30, 31]. Therefore, the ability of APF to halt cell proliferation and suppress the expression of genes involved in tumorigenesis is inhibited. Moreover, DHHC2 could stimulate palmitoylation of tetraspanins CD9 and CD151 [32]. CD9 is a potential tumor suppressor [33], while CD151 is supposed to promote tumor metastasis [34]. Our data together with these previous results support the notion that DHHC2 and its substrates may play an important role in the process of development, metastasis, and recurrence in various cancers including HCC. Despite several substrates have been identified, it is possible that more functional substrates remain unknown. Accordingly, our further study should focus on searching novel substrates and investigating the biological function of DHHC2 and its substrates in HCC, which may well provide novel and important targets for pharmacological intervention in the progression of HCC.

## 5. Conclusions

LOH was frequent on ZDHHC2, MCPH1, TUSC3, and KIAA1456 in human HCC. Of these, LOH on ZDHHC2 might contribute to early metastatic recurrence of HCC after LT. Reduced expression of ZDHHC2 was detected in HCC and associates with biological function of HCC such as proliferation, migration, and invasion. These results suggest an important role for ZDHHC2 as a tumor suppressor in metastasis and recurrence of HCC.

## Supplementary Material

Supplementary Table 1: RT-PCR primers.
Supplementary Table 2: Clinicopathological Correlation With ZDHHC2 mRNA expression in HCC Cases (Cohort 2).

## Figures and Tables

**Table 1:** Clinicopathological correlation with LOH in HCC cases (cohort 1).

Parameters	Genes	LOH/informative case		*P* value
Age		≤50 year	>50 year	
	ZDHHC2	12/17	5/21	0.004
	TUSC3	10/16	7/18	0.169
	MCPH1	11/17	9/20	0.231
	KIAA1456	10/16	6/15	0.210

Preoperative AFP level		≤400 ng/mL	>400 ng/mL	
	ZDHHC2	4/15	13/23	0.070
	TUSC3	5/12	12/22	0.473
	MCPH1	6/14	14/23	0.286
	KIAA1456	5/11	11/20	0.611

Tumor number		Single	Multiple	
	ZDHHC2	4/15	13/23	0.070
	TUSC3	5/12	12/22	0.473
	MCPH1	4/14	16/23	0.015
	KIAA1456	4/11	12/20	0.208

Tumor size		≤5 cm	>5 cm	
	ZDHHC2	6/23	11/15	0.004
	TUSC3	9/22	8/12	0.151
	MCPH1	9/22	11/15	0.052
	KIAA1456	8/18	8/13	0.347

PVTT		Absent	Present	
	ZDHHC2	11/31	6/7	0.031
	TUSC3	14/29	3/5	1.000
	MCPH1	14/30	6/7	0.097
	KIAA1456	11/24	5/7	0.394

Histopathologic grading		Well + moderately	Poorly	
	ZDHHC2	5/16	12/22	0.154
	TUSC3	3/11	14/23	0.067
	MCPH1	9/16	11/21	0.815
	KIAA1456	5/14	11/17	0.108

AFP: alpha-fetoprotein; PVTT: portal vein tumor thrombi.

**Table 2:** Univariate analysis of HCC recurrence.

Variables	*n*	1-year cumulative recurrence rate (%)	*P* value
LOH on ZDHHC2			
Positive	17	66.7 ± 12.2	0.022
Negative	11	30.0 ± 10.2	
Preoperative AFP level			
≤400 ng/mL	15	15.4 ± 10.0	0.005
>400 ng/mL	25	62.5 ± 9.9	
Tumor size			
≤5 cm	25	27.3 ± 9.5	0.001
>5 cm	15	73.3 ± 11.4	
PVTT			
Absent	33	35.5 ± 8.6	<0.001
Present	7	100	

AFP: alpha-fetoprotein; PVTT: portal vein tumor thrombi.

**Table 3:** Multivariate analysis of HCC recurrence.

Variables	Hazard ratio	95% confidence interval	*P* value
LOH on ZDHHC2	1.263	0.405–3.935	0.687
AFP >400 ng/mL	4.156	0.864–19.994	0.076
Tumor size >5 cm	1.615	0.430–6.063	0.478
PVTT (+)	3.468	1.004–11.982	0.049

AFP: alpha-fetoprotein; PVTT: portal vein tumor thrombi.
