# Supplementary material for: A Critical Role for ZDHHC2 in Metastasis and Recurrence in Human Hepatocellular Carcinoma
Source: Biomed Res Int. 2014 Jun 9;2014:832712. doi: 10.1155/2014/832712 (PMC4068081; doi:10.1155/2014/832712)
Supplement: Supplementary file 1 — Supplementary Table 1: RT-PCR primers. Supplementary Table 2: Clinicopathological Correlation With ZDHHC2 mRNA expression in HCC Cases (Cohort 2). [file 832712.f1.pdf]

Supplementary Table 1

RT-PCR primers

GAPDH forward	AGCCACATCGCTCAGACAC
GAPDH reverse	GCCCAATACGACCAAATCC
ZDHHC2 forward	TCTTAGGCGAGCAGCCAAGGAT
ZDHHC2 reverse	CAGTGATGGCAGCGATCTGGTT

Supplementary Table 2

Clinicopathological Correlation With ZDHHC2 mRNA expression in HCC Cases (Cohort 2)

Parameters	Tumor ZDHHC2 expression		*P*-value
	Low	High	
Age			
≤50 year	23	8	0.542
>50 year	16	8	
Preoperative AFP level			
≤400 ng/ml	18	9	0.551
>400 ng/ml	20	7	
Tumor number			
Single	24	8	0.431
Multiple	15	8	
Tumor size			
≤5 cm	20	13	0.039
>5 cm	19	3	
PVTT			
Absent	30	16	0.046
Present	9	0	
Histopathologic grading			
Well + moderately	20	4	0.074
Poorly	19	12	

AFP, alpha-fetoprotein; PVTT, portal vein tumor thrombi.
